# Enhancing Anti-G Antibody Induction by a Live Single-Cycle Prefusion F—Expressing RSV Vaccine Improves In Vitro and In Vivo Efficacy

**DOI:** 10.3390/v14112474

**Published:** 2022-11-09

**Authors:** Pramila Lamichhane, Megolhubino Terhüja, Timothy A. Snider, Antonius G. P. Oomens

**Affiliations:** Department of Veterinary Pathobiology, College of Veterinary Medicine, Oklahoma State University, Stillwater, OK 74078, USA

**Keywords:** respiratory syncytial virus, RSV, vaccine, attachment protein, G protein, prefusion F

## Abstract

The human respiratory syncytial virus (RSV) is a major cause of severe respiratory tract disease, and a vaccine is not available. We previously reported a novel live vaccine expressing prefusion-stabilized fusion protein (preF) in place of the native F protein (RSV-preF^ΔCT^). As preF is non-functional, RSV-preF^ΔCT^ was amplified in a production line expressing a functional substitute, and exhibited a single-cycle replication phenotype, which holds several unique potential advantages. RSV-preF^ΔCT^ prevented shedding and lung pathology after viral challenge in mice, but induced low levels of anti-attachment protein (G) antibodies (Abs). Given the significant contributions of anti-G Abs toward disease prevention, we generated modifications to RSV-preF^ΔCT^ in an effort to induce higher anti-G Ab levels. The Ab levels were monitored after the prime-boost vaccination of mice with modified vaccines. The most successful modification for enhancing induced anti-G Abs was seen with the placement of G in the first genome position. This vaccine also reduced the pathology after challenge with a high dose of wt RSV, and outperformed the sera from wt RSV-vaccinated mice in in vitro neutralization. Thus, raising the anti-G Ab levels induced by RSV-preF^ΔCT^ enhanced efficacy in vitro and in vivo, and constitutes an important next step in developing a live, single-cycle, efficacious vaccine for the human population.

## 1. Introduction

The human respiratory syncytial virus (RSV) is one of the most common respiratory tract pathogens of the pediatric population, and is also recognized as a pathogen of elderly and immunosuppressed individuals. Globally, an estimated 95,000–150,000 deaths occur in children < 5 years of age [1]. In the United States alone, an annual estimate of 132,000–172,000 RSV-associated hospitalizations of children, predominantly infants, have been reported [2]. Despite the substantial disease burden and decades-spanning efforts in vaccine development, there is no RSV vaccine available.

The antibody (Ab) response to RSV infection is primarily directed against two viral surface glycoproteins: the fusion protein (F) and the attachment protein (G). The RSV F protein is essential, as it mediates fusion between the virion and host cell membrane during viral entry. The membrane fusion process requires a conformational change in F from a highly unstable pre-fusion form (preF) to a stable post-fusion form (postF) [3]. This conformational change to the postF form may happen prematurely upon protein or virus manipulation or purification. The latter affects antigenic potential, as it has been shown that the majority of RSV-neutralizing Abs are directed against the preF conformation [3,4]. The RSV G protein is a highly glycosylated mucin-like protein. G is not essential for the infection of immortalized cell lines [5], but is required for the efficient infection in vivo, and the infection of primary well-differentiated human airway epithelial (HAE) cells [6,7,8,9,10]. When grown in Vero cells (one of several FDA-approved cell lines for vaccine production), the G protein was shown to be cleaved by cathepsin L (CatL), presumably due to recycling from the cell surface [11,12]. This resulted in reduced virion infectivity, suggesting that CatL cleavage compromises the attachment function of G [8]. A point mutation (L208A) that blocked CatL cleavage in Vero cells was identified, and this resulted in increased virion infectivity in HAE cells cultures [11]. In addition to a membrane-anchored virion-associated form (mG), a secreted form (sG) is produced by translation initiation at a second methionine codon (M48). sG is a known virulence factor as it acts as an antigen decoy, interferes with RSV clearance, and modulates immune cell functions [13,14,15]. For vaccine purposes, the role of sG is not clear as both detrimental [16] and protective effects [17] have been reported.

We previously generated a live vaccine, RSV-preF^ΔCT^, in which SH and the native F open reading frame (ORF) were removed and replaced with that of a DS-Cav1-based prefusion-stabilized membrane-anchored F protein (preF), and GFP [3,18,19]. To enhance the surface expression, and to ensure that F would not regain functionality when incorporated in a live virus, the F cytoplasmic tail (CT) was removed, and the resulting protein was termed preF^ΔCT^ [18,19]. The preF^∆CT^ gene was placed in the sixth genome position to further enhance expression, and a GFP ORF was placed in the natural F (eighth) position. In addition to changes in F, the G gene of RSV-preF^ΔCT^ was modified to ablate the expression of sG, which may serve as a virulence factor (for review see [20]). As preF was no longer functional, RSV-preF^ΔCT^ was recovered and amplified in Vbac cells that express a hybrid baculovirus GP64 protein, which serves as a functional substitute for F [21,22]. As a consequence, RSV-preF^ΔCT^ has a single-cycle replication phenotype, and cannot generate infectious progeny after the infection of normal cell types. In adult mice, the prime-boost intranasal vaccination with RSV-preF^ΔCT^ was found to induce high levels of anti-preF Abs with an increased ratio of preF:postF Abs and antiviral T cells. Although immune responses elicited by RSV-preF^ΔCT^ did not completely neutralize wt RSV in vitro, mice were protected from lung pathology and viral shedding after challenge with a high dose of wt RSV. Thus, in protecting mice, presenting only preF antigen, and blocking progeny virus production, RSV-preF^ΔCT^ possesses unique safety and efficacy advantages.

A surprising finding emerged: despite containing the mG ORF, RSV-preF^ΔCT^ induced only very low levels of anti-G Abs [18]. A large body of work has shown the importance of anti-G Abs in the protection against pathology and the reduced severity of RSV disease in animal models and human infants [23,24,25,26,27,28,29,30,31,32,33]. For example, treatment with anti-G Abs may reduce pulmonary inflammation, inflammatory cell numbers, viral load, lung Th-2 cytokine levels, mucus levels, and breathing effort (for review see [23]). Moreover, anti-G Abs also avoid the dependence on F as a singular surface antigen. Furthermore, since RSV-preF^ΔCT^ replicates only for one round, we anticipate that we will need to maximize its efficacy potential to consider future application in humans. Our first priority was to attempt to increase the level of anti-G Abs induced by a single-cycle preF-expressing live vaccine.

To do so, we made several genetic modifications to RSV-preF^ΔCT^ and examined the impact of these modifications on G and F specific Ab levels using a mouse model. The prevention of premature cleavage of G by CatL, the re-introduction of sG, or the use of vaccine viruses that separately expressed G or preF, all failed to raise the anti-G Ab levels in vaccinated mice. In contrast, increasing input levels of G by including a G protein-containing VLP, or by moving the G ORF to the first position within the genome, resulted in a vaccine that significantly raised the anti-G Ab levels. Serum Abs induced by the latter vaccine significantly lowered lung pathology induced by a high-pathogenic wt RSV, and also neutralized wt RSV in vitro significantly better than serum from those mice similarly vaccinated with wt RSV or RSV-preF^ΔCT^. In short, moving the G ORF to the first position of RSV-preF^ΔCT^ led to a single-cycle virus that outperformed wt RSV in its ability to induce neutralizing and protective Abs after vaccination.

## 2. Materials and Methods

### 2.1. Cells and Antibodies

HEp-2 cells (American Type Culture Collection) were maintained in advanced Dulbecco’s Modified Eagle medium (DMEM) supplemented with: 4% fetal bovine serum; 50 units/mL penicillin; 50 μg/mL streptomycin; and 2 mM glutamax. Vbac cells expressing chimeric glycoprotein GP^64/F^ (baculovirus GP64 with RSV F CT) were previously generated [22] and were maintained in the same medium plus 0.2 mg/mL G418 every other passage. Monoclonal antibodies (MAb) D25 was kindly provided by Jason McLellan (University of Texas at Austin, Austin, TX, USA). MAb L9 [34] was provided by Ed Walsh (University of Rochester School of Medicine, Rochester, NY, USA). Rabbit polyclonal anti-M and anti-F peptide sera were reported previously [18,35]. Anti-N antibody was acquired from AbD Serotec. The anti-myc antibody was acquired from the Developmental Studies Hybridoma Bank, created by the NICHD of the NIH. Anti-flag Ab was acquired from Genscript.

### 2.2. Construction of Recombinant RSV cDNAs

To modify G, various point mutations were introduced by site-directed mutagenesis into a plasmid containing a non-codon-optimized A2 strain wt G ORF. To remove secreted G expression, a point mutation was introduced that resulted in M48L (designated mG). To prevent G cleavage by CatL, point mutation L208A [11] was introduced in mG. To modify serine 2, a silent mutation was introduced in mG (TCC to AGC). To insert the modified G ORFs into an RSV cDNA, the original G ORF was removed and replaced with a cloning cassette containing two BsmBI sites. Modified G constructs were then PCR amplified and cloned into G-deleted cDNA using BsmBI. No foreign sequences other than the intended modifications were introduced. To remove the preF^ΔCT^ or mG ORF from RSV-preF^ΔCT^, these ORFs were removed and replaced with chloramphenicol transferase (CAT)-based spacers to maintain the same total number of ORFs. To generate A2/K191R, an F-containing plasmid was mutated using site-directed mutagenesis. The resulting F variant, F/K191R, was then cloned using conventional techniques into a wt RSV cDNA lacking the F ORF and containing, instead, a cloning cassette with BsmBI sites. The construction of an HRP-expressing RSV (RSV-HRP) was described previously [18].

### 2.3. Recovery of Viruses from cDNA and Production of Virus Stocks

Infectious preF-expressing viruses were recovered from cDNA as described previously [18,35,36] with minor modifications. Briefly, BHK-21 cells expressing T7 polymerase were transfected with engineered cDNAs and internal ribosome entry site-containing support plasmids expressing nucleoprotein N, phosphoprotein P, transcription elongation factor M2–1, and polymerase L. In addition, a plasmid-expressing baculovirus GP64 containing an F CT (GP^64/F^) was included to enable a progeny virus generated in the transfected cells, to infect Vbac cells [21,22]. After incubation at 33 °C for 70 h, supernatant from the transfected cells was transferred to Vbac cells and incubated for another seven days at 33 °C. Cells were harvested by the scraping and removal of cell debris by centrifugation at low g-force. These stocks were frozen at −80 °C as low-passage seed stocks. All virus stocks used in the experiments were generated directly from these seed stocks. To generate vaccine batches, Vbac cells were infected with seed stock and incubated at 33 °C for seven to eight days. Virus was harvested by scraping cells, pipetting the solution vigorously, and removing cell debris by low-speed centrifugation. Virus was then pelleted by centrifugation at 40,000× *g* through a 25% sucrose cushion. Batches of previously described surrogate wt virus RSV-rWT [36] (used as a vaccination control) and recombinant wt A2/K191R virus (used for challenge studies) were similarly produced with the exception that partial purification through a sucrose cushion was conducted at 5000× *g*. After centrifugation, virus pellets were re-suspended in OPTIMEM containing 5% sucrose and 100 mM MgSO4, flash-frozen in liquid nitrogen, and stored at −80 °C. Virus stocks were titrated in duplicate by plaque assay (plaques scored by observing GFP-expression on day 3–5), and the genome of engineered viruses was confirmed by bulk sequencing of modified genome areas after purifying viral RNA from infected cells and RT-PCR.

### 2.4. Generation of VLP-G

To generate VLPs containing the G protein, HEK-293 Freestyle cells were used in shaker cultures in Freestyle 293 Expression medium (Invitrogen, Waltham, MA, USA). Cells were transfected for 5 h with plasmids expressing RSV P (containing a C-terminal flag tag), M, Fstem (F with residues 36–495 deleted and containing a myc epitope for detection [37]), and G proteins. At 22 hpt, Freestyle medium was replaced with OPTIMEM. At 44 hpt, the cells were removed by low-speed centrifugation. VLPs were semi-purified from the supernatant by ultracentrifugation through a 25% sucrose cushion. The pellet was washed and then resuspended in PBS. The total protein concentration in the VLPs was quantified using a Micro BCA Protein Assay Kit (Thermo Fisher Scientific, Waltham, MA, USA).

### 2.5. Cell ELISA to Measure preF and G Levels on the Surface of Infected Cells

The surface expression was examined in infected HEp-2 cells at 26 hpi, as previously described with minor modifications [18,21,37]. Primary Abs were incubated on unfixed cells (to maintain native conformation) for 2 h. After washing away unbound Abs, cells were fixed for 5 min with freshly dissolved 4% paraformaldehyde. This was followed by incubation with a horseradish peroxide-conjugated (HRP) secondary antibody and washing steps. After the last wash step, the cells were incubated in O-phenylenediamine dihydrochloride (OPD)(Thermo Fisher Scientific, Waltham, MA, USA)-based ELISA substrate. At short time intervals, aliquots were taken and added to 3M sulfuric acid in a 96-well plate to stop the reaction. The optical density at 490 nm (OD_490_) was measured in a Versamax plate reader (Molecular Devices, San Jose, CA, USA). For the normalization of infected cells, N protein was measured by similar fixation and permeabilization with 0.2% triton, prior to its incubation with anti-N antibody. Error bars indicate the standard deviation from the mean of triplicate samples. Each experiment was carried out twice independently with similar results. The statistical differences were determined by unpaired two-tailed Student’s *t*-tests with Welch’s correction (Prism version 9.4.1, Graphpad Software LLC, San Diego, CA, USA).

### 2.6. Western Blot Analysis

For the detection of sG , vero cells were infected at high moi in a six-well plate and supernatant was harvested at 26 hpi. Virus was removed by centrifugation (60 min, 21,000× *g*). sG was immuno-precipitated from the supernatant using L9 and magnetic protein G beads (Dynabeads) (Invitrogen, Waltham, MA, USA). The final pellets were washed four times and then boiled in Laemli buffer. Mock-infected cell supernatant was included in the immuno-precipitation as a negative control; RSV-rWT infected cells were included as a positive control for sG expression. L9 was used to detect G protein. Because the same Ab was used for immuno-precipitation and detection on the blot, the heavy and light chains of L9 are also visible on the Western blot. To detect G in VLP-G, semi-purified VLP-G were mixed with Laemli buffer and boiled. A lysate of cells infected with wt RSV was included as a positive control; an uninfected 293 cell lysate served as a negative control. The following primary Abs were used for protein detection: L9 (to detect G); anti-M rabbit serum (to detect M); anti-myc Ab (to detect Fstem); anti-FLAG (to detect P). To detect G and F in virions, viruses were pelleted (60 min, 21,000× *g*), resuspended, and boiled in Laemli buffer. Equivalent PFUs (100,000 PFU/lane) of the viruses were compared. Western blots were first probed with anti-F and anti-M peptide sera, then stripped and re-probed with anti-M serum and L9. In all cases, 12% SDS-PAGE gels were used, and proteins were transferred to Immobilon blots using a semi-dry apparatus (Bio-Rad, Hercules, CA, USA). Blots were developed using enhanced chemiluminescence and scanned on an Amersham Imager 600 (GE). Each experiment was carried at least twice independently. The quantitation of G and F was performed using ImageJ software V 1.53 (NIH, Bethesda, MD, USA), and ratios of F:M and G:M were calculated.

### 2.7. Mouse Ethics Statement

Female BALB/c mice were purchased from Jackson Laboratory (Bar Harbor, ME, USA). All mouse studies were approved by the Institutional Biosafety Committee and the Institutional Animal Care and Use Committee at Oklahoma State University (OSU Stillwater animal assurance number: A3722-01). The experiments were performed under strict accordance to the Office of Laboratory Animal Welfare guidelines and the Public Health Service Policy on Humane Care and Use of Laboratory Animals.

### 2.8. Vaccination Protocol and Serum Ab Collection

Eight-week-old female mice were anaesthetized by intraperitoneal injection of 100 mg/kg of body weight for ketamine and 5 mg/kg of xylazine. The mice received prime and boost IN vaccinations three weeks apart, each consisting of 0.5 × 10^6^ PFU in a 50 µL volume. For vaccinations in which VLP-G was added, 0.5 × 10^6^ PFU was combined with 15 μg of VLP-G in a total volume of 50 µL. For the combination vaccine, 0.5 × 10^6^ PFU each of RSV-∆F and RSV-preF-∆G virus were combined into 50 µL. The mock vaccine group received an equal volume of material identically prepared from uninfected Vbac cells. At the 21-day post-boost point, blood samples were collected for serum Ab studies, using ELISA (see below).

### 2.9. Challenge Studies (Histopathology)

For challenge studies, mice were vaccinated as above, followed by an IN challenge with 2 × 10^6^ PFU of A2/K191R, four weeks after boost vaccination. A2/K191R stock was grown in HEp-2 cells. The mock-challenge consisted of material identically prepared from uninfected HEp-2 cells. Five days post challenge, mice were humanely sacrificed, and lungs were collected for histopathology. Lungs were gently inflated with 10% neutral buffered formalin, and then immersed in the same fixative. Lungs were routinely processed through graded alcohols and xylene, embedded in paraffin, sectioned at 4 µm, and stained with hematoxylin and eosin (H&E). Slides were examined blindly by an ACVP-certified veterinary pathologist and scored for six parameters (see Section 3) using a scale of 0 (no pathology) to 3 (high pathology). The average total pathology scores were determined for each group (*n* = 5) by adding up all parameter scores from individual mice and then dividing by five. Statistical significance was determined by unpaired two-tailed Student’s *t*-tests with Welch’s correction.

### 2.10. ELISA to Measure Anti-preF and Anti-G Ab Levels in Mouse Sera

To coat antigens to the plate, nickel-coated plates were incubated with his-tagged purified preF, postF, or G proteins. Purified preF and postF proteins were kindly provided by Jason McLellan (University of Texas at Austin, Austin, TX, USA). Baculovirus produced purified G protein (residues 67 to 298; roughly corresponding to the ectodomain) was commercially acquired (MyBioSource 1059692). Coated plates were washed, blocked with 5% milk powder (Bio-Rad, Hercules, CA, USA), and incubated with mouse sera (pooled from a total of 8–10 mice/group from two independent experiments) diluted in OPTIMEM in three-fold steps with a starting concentration of 1:100. Primary Abs were incubated for two hours at room temperature, followed by wash steps and incubation with HRP-conjugated secondary Ab. Plates were washed three times, and developed using OPD substrate as above. The error bars indicate standard deviation from the mean of triplicate samples from the pooled sera. Each experiment was carried twice independently. Statistical differences between the curves were determined as follows: OD values of selected dilutions were directly compared by unpaired two-tailed Student’s *t*-tests with Welch’s correction. The differences were determined only when dilutions fell within the linear range of the curves. Statistical significances indicated in the graphs mean that at least two dilutions (from the linear part of the curve) that were tested, were significantly different. In most cases, only statistical differences between the parent vaccine (RSV-preF^∆CT^) and modified vaccines were determined.

### 2.11. In Vitro Neutralization

Mouse serum samples of each group were pooled and heat inactivated at 56 °C for 30 min. Random mouse IgG (Pierce, Waltham, MA, USA) and sera from mock-vaccinated mice were included as negative controls. The three-fold serial dilutions of samples were prepared in a medium containing heat-inactivated FBS. Next, 200 PFU of RSV-HRP were mixed with the diluted mouse sera, incubated for 1 h at 37 °C, and transferred to HEp-2 monolayers. Virus was adsorbed for 1 h and then replaced with fresh medium. Following incubation for 48 h, the supernatant was replaced by 3,3′,5,5′-tetramethylbenzidine solution (Thermo Fisher Scientific, Waltham, MA, USA), incubated for 30 min, and the reaction was stopped by adding 2M sulfuric acid. The optical density at 450 nm was measured. The experiment was performed twice from two independent mouse studies with *n* = 5 each, with similar results. Error bars represent the standard deviation of the mean of triplicate samples from the pooled sera. Curve-fit analyses were performed using Graphpad Prism 9 and reciprocal titers that achieved 50% neutralization of the RSV-HRP were calculated. A reliable curve fit for RSV-preF^∆CT^ could not be performed due to the incomplete neutralization. Significances were determined by unpaired two-tailed Student’s *t*-test with Welch’s correction.

## 3. Results

### 3.1. Overview of Approaches Aimed at Enhancing Serum Anti-G Ab Levels Induced by a Single-Cycle Pref-Expressing Virus

**Figure 1:**
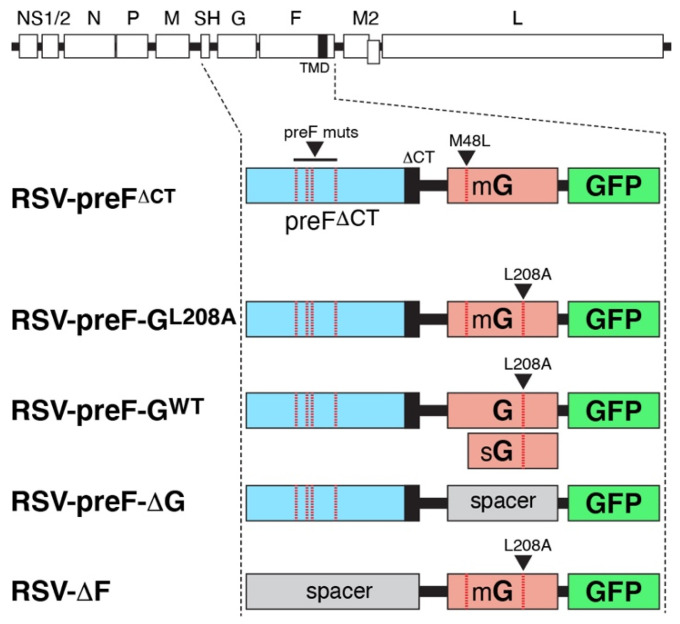
Overview and composition of modified viral genomes. A schematic of the wt RSV genome is shown on top, followed by the composition of previously reported virus RSV-preF^ΔCT^ [18]. In the latter, the SH, G, and F ORFs were replaced, respectively, by ORFs encoding preF^∆CT^ (a membrane-anchored prefusion-stabilized F based on DS-Cav1 mutations and lacking its CT), a membrane-anchored G protein (mG) based on M48L mutation, and GFP. RSV-preF^ΔCT^ was used as a comparison throughout but was also used in combination with G-containing VLPs (see later). In RSV-preF-G^L208A^, an L208A mutation was made in G to block premature CatL cleavage in our Vero-derived Vbac production cell line. In RSV-preF-G^WT^, the codon for methionine 48 was restored resulting in expression of both mG and sG. RSV-preF-G^WT^ also contains the mutation L208A. In RSV-preF-∆G and RSV-∆preF, either the G or preF^ΔCT^ ORF was removed and replaced with a spacer sequence (spacers are not drawn to scale). The latter two viruses were used together as a combination vaccine. An additional genetic approach in which the G gene was rearranged is described below. All viruses were recovered from cDNA and verified as previously described, and amplified in Vbac cells [18]. Dotted red lines and arrowheads indicate the location of DS-Cav1, M48L, or L208A mutations.

As described above, our previously reported virus RSV-preF^ΔCT^ showed unique safety and efficacy potential. In spite of expressing wt levels of G on the surface of in vitro infected cells, RSV-preF^ΔCT^ was a poor inducer of anti-G Abs in mice. The unexpected low anti-G Ab levels after vaccination may compromise the efficacy of a single-cycle vaccine in humans, and leave Ab-dependent immunity based on a single surface antigen (F). Our first priority, therefore, was to attempt to increase the level of anti-G Abs induced by RSV-preF^ΔCT^. To accomplish the latter, several distinct genetic modifications were made. An overview of these modifications is shown in Figure 1. Further details on the modifications, and their rationales, are described in the individual result sections below. One additional approach involving the G gene rearrangement is shown below. All viruses were recovered from cDNA and amplified in Vbac cells as previously described [21,22].

### 3.2. A mutation That Blocks CatL Cleavage Does Not Raise G Ab Levels in Mice Vaccinated with RSV-preF^∆CT^

**Figure 2:**
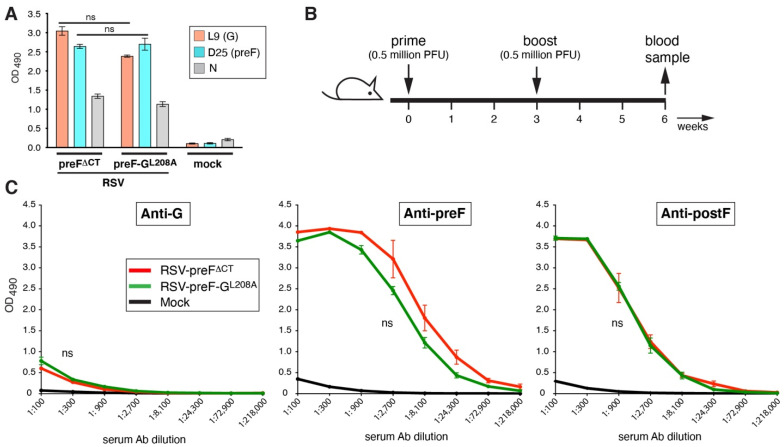
Impact of the L208A mutation in G on the ability of RSV-preF^∆CT^ to induce anti-G Abs. (**A**) F and G protein expression on the surface of virus-infected cells by cell ELISA. Hep2 cells were infected with RSV-preF^∆CT^, RSV-preF-G^L208A^ or mock-infected. At 26 hpi, unfixed infected cells were incubated with either D25 (preF-specific Ab) and L9 (G-specific Ab), washed, fixed, and incubated with secondary Ab to determine surface level by ELISA. N protein level was determined in parallel fixed and permeabiized cells as an infection control. (**B**) Vaccination and sampling schedule. Eight-week-old BALB/c mice were intranasally vaccinated 2× three weeks apart with 0.5 million PFU/animal. Blood samples were taken three weeks post-boost. Mock vaccine was generated exactly as the experimental vaccines but from uninfected Vbac cells. (**C**) Serum levels of G-, preF-, and postF-specific IgG Abs induced in vaccinated mice. Three-fold dilutions of pooled boost sera from mice from two independent experiments (total *n* = 9/group) were incubated on plates coated with purified G, preF, or postF proteins, and Ab levels were determined by ELISA. Error bars represent the standard deviation of the mean of triplicate samples from pooled sera. In (**C**), only statistical differences between RSV-preF^ΔCT^ and RSV-preF- G^L208A^ were determined (ns, non-significant).

In contrast to HEp-2 or HeLa cells, Vero cells were previously shown to have very high CatL activity, and to cleave the G protein between residues 209 and 210 [11]. This cleavage was shown to severely compromise the attachment function of G in human airway epithelial cells [8] and may impact the kinetics of live vaccines produced in Vero cells. Our production cell line used for the amplification of RSV-preF^ΔCT^ (Vbac) is a Vero-derived cell line. Hence, we reasoned that the potential cleavage of G during the production of RSV-preF^ΔCT^ may have contributed to the low anti-G levels induced by this virus in mice. A previously reported mutation that blocks CatL cleavage in Vero cells (leucine 208 to alanine, [L208A]) [11], was introduced into a cDNA of RSV-preF^ΔCT^ and a virus was recovered and termed RSV-preF-G^L208A^. Next, we examined whether L208A impacted the G expression levels at the surface by cell ELISA using anti- GAb (L9). L9 [34] recognizes the CX3C region which is present in both cleaved and uncleaved versions of G (Figure 2A). This was examined in HEp-2 cells to better compare the two viruses, and L9 was incubated on cells prior to fixation to maintain G conformation, as previously described [18]. An anti-nucleoprotein (N) Ab was used on separate wells with fixed/permeabilized cells to verify similar rates of infection, and a preF-specific Ab D25 was used to simultaneously measure preF levels. At 26 hpi, the two viruses induced near-identical levels of G and preF protein (at the cell surface) and N (internal), indicating that L208A did not have unintended deleterious effects on viral replication or protein expression.

We next compared the G and F Ab levels induced by RSV-preF^∆CT^ and RSV-preF-G^L208A^ in mice to examine whether the L208A mutation increased the Ab levels after vaccination. Eight-week-old mice were prime-boost vaccinated intranasally using 0.5 million PFU per dose, as shown in Figure 2B and as previously described [18]. A mock-vaccinated group was included in which the mock vaccine was prepared identically from uninfected Vbac cells. Three weeks after the boost, blood samples were taken, and the Ab levels were determined by ELISA (Figure 2C). In addition to anti-G Abs, we also measured the anti-F Ab levels to ensure that any changes made in G would not negatively impact the anti-F Ab levels. This was conducted using purified his-tagged G, preF, and postF antigens as previously described and validated [18]. The absence of non-specific binding by mouse sera was confirmed in Figure S1. The ELISA results show equal levels of anti-G, -preF, -postF Abs between the two viruses (Figure 2C). Thus, premature CatL cleavage was not a main cause of low anti-G Ab levels induced by virus RSV-preF^∆CT^.

### 3.3. The Impact of Re-Introducing sG on Anti-G Ab Levels in Vaccinated Mice

**Figure 3:**
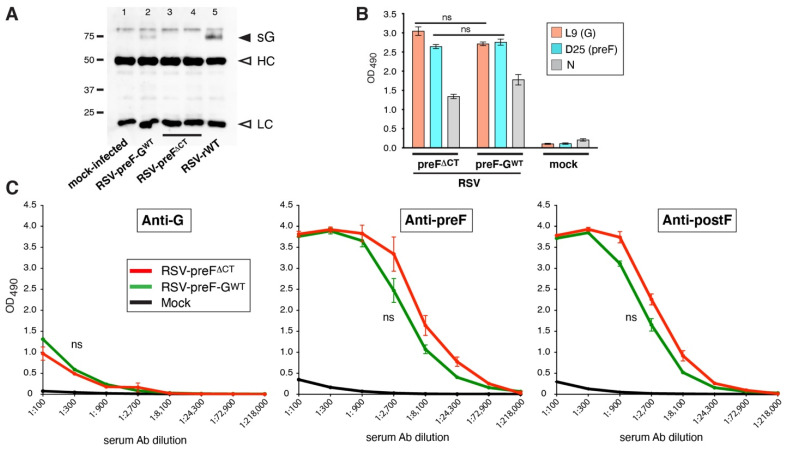
Impact of restoring sG expression on the ability of RSV-preF^∆CT^ to induce anti-G Abs. (**A**) sG expression by RSV-preF-G^WT^. HEp-2 cells were infected with the viruses indicated. RSV-rWT, a previously reported surrogate wt virus [36], was included as a positive control for sG expression. Supernatants at 26 hpi were centrifugated at high g-force to remove virus particles and immunoprecipitated using Ab L9 as described in Materials and Methods. A Western blot was generated in which G was detected using Ab L9. Open arrows indicate L9 heavy chain (HC) and light chain (LC) recognized by the goat-anti-mouse HRP secondary. (**B**) F and G protein expression on the surface of virus-infected cells by cell ELISA, as described in the Figure 2 legend. (**C**) Serum levels of G-, preF-, and postF-specific IgG Abs induced in mice, vaccinated, as described in Figure 2. Three-fold dilutions of pooled boost sera from mice from two independent experiments (total *n* = 8/group) were examined by ELISA, as described in Figure 2. Error bars represent the standard deviation of the mean of triplicate samples from pooled sera. In (**C**), only statistical differences between RSV-preF^ΔCT^ and RSV-preF-G^WT^ were determined (ns, non-significant).

In RSV-preF^∆CT^, the expression of sG was ablated because several reports have described detrimental immune-modulatory effects by sG (for review see [20]). Here, we questioned whether the absence of sG may have contributed to the low anti-G Ab levels induced by RSV-preF^∆CT^. In line with a potentially beneficial role for sG in live vaccines is a recent study by Liang et al. [17]. In this study, the authors found a modest improvement in protection in hamster nasal turbinates, after comparing a chimeric bovine/human parainfluenza virus type 3 (rB/HIPV3) vaccine with and without sG. To address the potential role for sG in anti-G Ab induction by RSV-preF^∆CT^, we modified the G ORF of RSV-preF^ΔCT^ to one that expresses both mG and sG, and amplified the resulting virus, termed RSV-preF-G^WT^, in Vbac cells. RSV-preF-G^WT^ also has the L208A mutation in G (which was shown not to impact G Ab levels, see Figure 2), to avoid potential CatL cleavage of sG. First, we verified that sG expression was restored in RSV-preF-G^wt^ (Figure 3A). Cells were infected with the latter virus or with RSV-preF^∆CT^, or with the surrogate wt RSV (RSV-rWT) previously described [35,36].

Supernatant from infected cells was harvested at 26 hpi, clarified, and sG immuno-precipitated with anti-G Ab L9. A Western blot revealed that, as expected, RSV-preF^∆CT^ lacked sG expression (Figure 3A, lanes 3, 4), whereas RSV-preF-G^WT^ and RSV-rWT (Figure 3A, lanes 2, 5) showed a high Mr band consistent with sG. We also verified viral protein surface expression levels by cell ELISA, as above, using the same infected cell samples used for the sG secretion analysis (Figure 3B). The N protein was again included to show differences in the rate of infection. The two viruses showed similar preF and G expression levels, suggesting that the inclusion of sG does not cause major differences in viral replication or protein expression.

Next, we examined whether the inclusion of sG could raise the anti-G Ab levels in vaccinated mice, by determining the serum’s Ab levels by ELISA at three weeks post-boost, as above (Figure 3C). For both viruses, a similar very low level of anti-G Abs was observed. Thus, the inclusion of sG did not enhance anti-G Ab induction in mice, and is not a cause of low anti-G Ab levels induced in RSV-preF^∆CT^ vaccinated mice. The anti-preF and -postF Ab levels also remained unchanged.

### 3.4. Presenting preF and G on Separate Vaccine Particles Does Not Improve G or F Ab Levels in Mice

**Figure 4:**
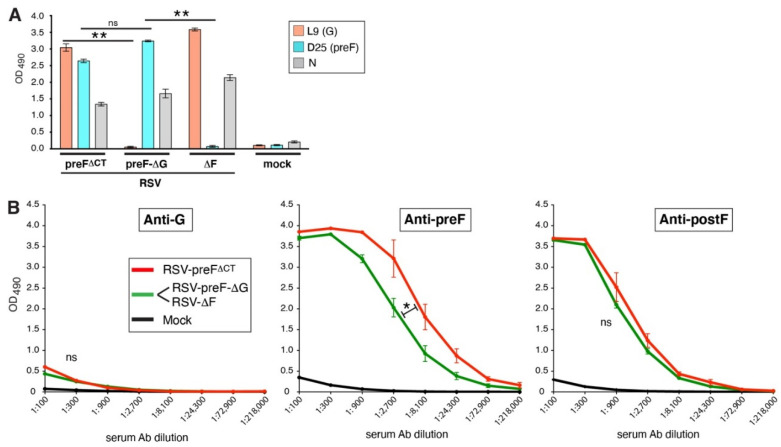
Presenting G and preF on separate particles in a combination vaccine does not induce high levels of anti-G Abs. (**A**) F and G protein expression on the surface of virus-infected cells by cell ELISA, as described in the Figure 2 legend. Although RSV-preF-ΔG and RSV-ΔF were used as a combination vaccine in part B, they were separately examined to verify the lack of G and F expression, respectively. (**B**) Serum levels of G-, preF-, and postF-specific IgG Abs induced in mice vaccinated, as described in Figure 2. For the combination vaccine, prime and boost vaccination consisted of 0.5 million of RSV-preF-ΔG and RSV-ΔF viruses. Three-fold dilutions of pooled boost sera from mice from two independent experiments (total *n* = 8/group) were examined by ELISA, as described in Figure 2. Error bars represent the standard deviation of the mean of triplicate samples from pooled sera. In (**B**), only statistical differences between RSV-preF^ΔCT^ and the combination vaccine were determined. (* *p* < 0.05; ** *p* < 0.01; ns, non-significant).

In yet another approach to raise the level of anti-G Abs, we generated viruses separately expressing G or preF. This approach examined whether one of the two surface antigens in our vaccine might exert dominance over, or be shielded by, the other, when co-present in the same virion. A cDNA representing RSV-preF^∆CT^ was modified by replacing the G or the preF ORF by a spacer. The resulting viruses were amplified in Vbac cells and termed RSV-preF-∆G and RSV-∆F, respectively. Viral protein expression was examined by cell ELISA, as above, and confirmed that these viruses lacked G or preF expression (Figure 4A). To examine the impact of G and preF separation on the Ab levels in mice, equal PFUs (0.5 million) of RSV-preF-∆G and RSV-∆F virus were combined to vaccinate mice, as described above, and sera from vaccinated mice were compared to sera from RSV-preF^∆CT^ vaccinated mice (Figure 4B). The ELISA results showed that the anti-G and anti-postF Ab levels did not change. The anti-preF Ab levels were, however, moderately lower (*p* < 0.05) in mice vaccinated with the combined vaccine. In short, the separation of the G and preF antigens did not raise the anti-G Ab levels, and instead caused an undesirable impact on the preF Ab levels.

### 3.5. Addition of Exogenous Input G Protein Increases Anti-G Ab Levels in Vaccinated Mice

**Figure 5:**
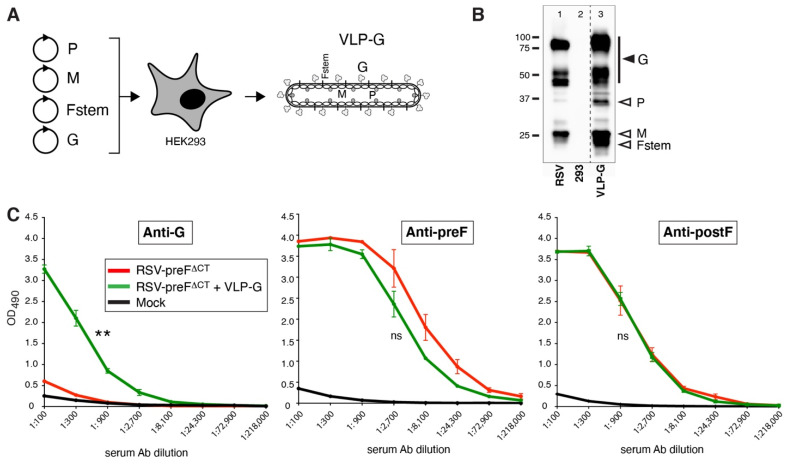
The impact of exogenous G on the ability of RSV-preF^∆CT^ to induce anti-G Abs. (**A**) Schematic of VLP-G production. Plasmids expressing P, M, Fstem, and G were co-transfected in HEK293 cells as described in Materials and Methods, and VLPs were semi-purified. (**B**) Protein content of VLP-G. VLP-G made in HEK293 cells were harvested and semi-purified as described in Materials and Methods, pelleted, and examined by Western blot. A plasmid expressing Fstem protein (Fs), which lacks the F ectodomain (residues 36–495) and was included to enhance VLP production, was described previously [37]. The equivalent of 9 µg VLP-G was loaded in one lane. As a comparison, lysate of wt RSV infected cells was included (~7500 cells/lane) as a positive control for G. Primary Abs used for detection were as follows: L9 (G); anti-M peptide serum [35] (M); anti-flag Ab (N and P have flag tags); anti-myc Ab (Fs has a myc tag). Note: the dotted line shows where several lanes were deleted, and the images re-joined; image intensity or appearance was not altered. (**C**) Serum levels of G-, preF-, and postF-specific IgG Abs induced in mice vaccinated, as described in Figure 2. Three-fold dilutions of pooled boost sera from mice from two independent experiments (total *n* = 5/group) were examined by ELISA as described in Figure 2. Error bars represent the standard deviation of the mean of triplicate samples. In (**C**), only statistical differences between RSV-preF^ΔCT^ and RSV-preF^ΔCT^ + VLP-G were determined (** *p* < 0.01; ns, non-significant).

The relative contribution of input versus the de novo synthesized G to the anti-G Ab induction by a live vaccine is not known, and will also depend on the level of genomic replication. We previously reported that the low amount of G protein in RSV-preF^∆CT^ vaccine particles (input G) was a potential cause of the low level of serum anti-G Abs induced by RSV-preF^∆CT^ in mice [18]. Here, we examined the importance of input G by including exogenous G protein in the IN vaccinations. A virus-like particle (VLP) was generated by transiently co-expressing P, M, G, and Fstem proteins in HEK-293 cells (see Materials and Methods), and termed VLP-G (Figure 5A). The plasmid expressing Fstem, which lacks the majority of the F ectodomain (residues 36–495), was described previously and shown to enhance VLP formation [37]. Partially purified VLP-G were characterized by Western blot, in which cell lysates from wt RSV infected HEp-2 cells and uninfected HEK293 cells were used as positive and negative controls, respectively (Figure 5B, lanes 1 and 2). The analysis confirmed the presence of G in the VLPs (Figure 5B, lane 3). To examine the impact of exogenous input G protein on anti-G Ab induction in mice, 15 µg of VLP-G was added to the IN prime and boost vaccinations with RSV-preF^∆CT^, and compared to the vaccination with RSV-preF^∆CT^ without added VLP-G (Figure 5C). The examination of vaccinated mouse sera showed, for the first time, a large increase in anti-G Abs, whereas no significant changes were observed in the anti-preF and -postF Ab levels. The addition of VLP-G to RSV-preF^∆CT^ is not likely to be a cost-effective vaccine strategy. Nevertheless, this indicates that the low G input level in the RSV-preF^∆CT^ vaccine particles may have contributed to low serum anti-G Ab levels in RSV-preF^∆CT^ vaccinated mice, and that adding additional input G protein may raise the anti-G Ab levels induced by a live vaccine.

### 3.6. The Impact of Moving G to the First Position in the Genome

**Figure 6:**
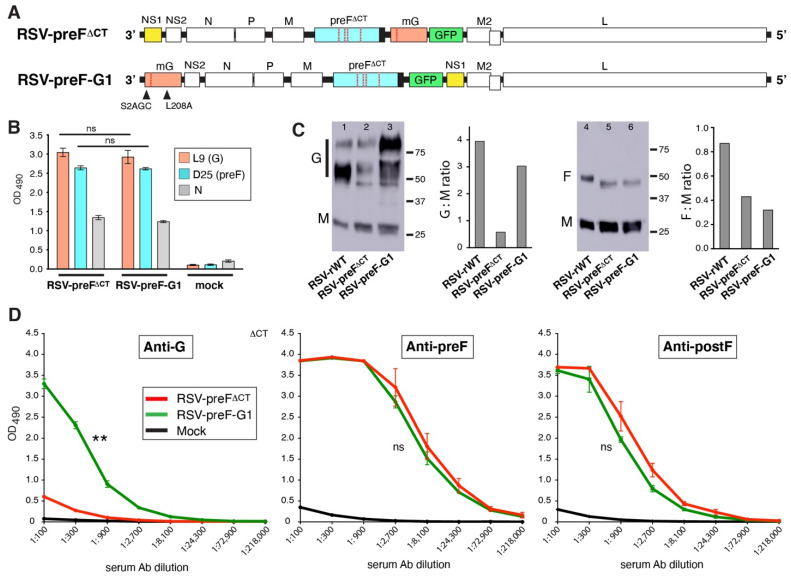
Composition, characterization, and analysis of a modified single-cycle preF-expressing virus with the G ORF at the first genome position. (**A**) Composition of RSV-preF-G1. RSV-preF-G1 differs from its parent vaccine in having mG and NS1 swapped such that mG is in first position, GFP at seventh position, and NS1 at eighth position. In addition, mG has the L208A mutation and has G residue 2 (serine) changed to AGC. (**B**) Expression of preF and G proteins on the surface of virus infected cells. Cells were infected with RSV-preF^∆CT^, RSV-preF-G1 or mock-infected, and G and preF surface levels were determined at 26 hpi using cell ELISA, as described above. (**C**) G and F protein content of vaccine viruses. Semi-purified virus particles were collected by pelleting at high g force and analyzed by Western blot (100,000 PFU/lane). M and F were detected by polyclonal peptide sera previously described [18,35]; G was detected by Ab L9. (**D**) Serum levels of G-, preF-, and postF-specific IgG Abs induced in vaccinated mice. Three-fold dilutions of pooled boost sera from mice from two independent experiments (total *n* = 10/group) were examined by ELISA, as described in Figure 2. Error bars represent the standard deviation of the mean of triplicate samples. In (**D**), only statistical differences between RSV-preF^ΔCT^ and RSV-preF-G1 were determined (** *p* < 0.01; ns, non-significant).

The last approach we pursued, in an effort to enhance serum anti-G Ab induction, focused on increasing G expression. As a gradient of gene expression exists with high expression of the first genome location, we modified RSV by moving the G ORF to position 1 (Figure 6A). To accommodate G at the first position, the NS1 ORF was moved to the eighth genome location, and the resulting virus was termed RSV-preF-G1. Exchanging these genes rather than placing an additional gene at the first position maintains normal gene expression levels (except for NS1). Since NS1 is known to counter the IFN response, downregulating NS1 by placing it further downstream has potentially additional advantages. The focus of this effort, however, was to raise G expression. The G gene also contained the L208A mutation and the codon for serine 2 was changed from TCC to AGC (a non-coding change) to, potentially, further improve the translation context [38]. First, we examined the preF and G surface expression levels by infecting cells with RSV-preF^∆CT^ and RSV-preF-G1, and performing a cell ELISA as above using N protein levels as a control for the equal infection rates. Against expectation, relative G levels were identical between the two viruses (Figure 6B). We also examined the G and preF content of semi-purified particles by Western blot (Figure 6C). This showed that F levels were similar between RSV-preF^∆CT^ and RSV-preF-G1. In contrast, for G, we found that in spite of the similar surface levels observed in Figure 6B, RSV-preF-G1 particles contained higher levels of G than RSV-preF^∆CT^ particles. The G protein in RSV-preF-G1 particles also displayed a higher proportion of mature 90–100 kD protein than RSV-rWT and RSV-preF^∆CT^ particles. This could be explained by the presence of mutation L208A in RSV-preF-G1. Next, we examined whether moving G to the first position might increase the anti-G Ab levels in vivo. Mice were vaccinated with RSV-preF-G1 or RSV-preF^∆CT^, and post- boost sera were examined by ELISA, as above (Figure 6D). While the anti-preF and -postF Ab levels were unchanged, the anti-G Ab levels were substantially increased, showing the feasibility of increasing the anti-G Ab levels without adding exogenous G protein as in Figure 5.

### 3.7. Parameters Contributing to Enhanced Anti-G Ab Levels Induced by RSV-preF-G1

**Figure 7:**
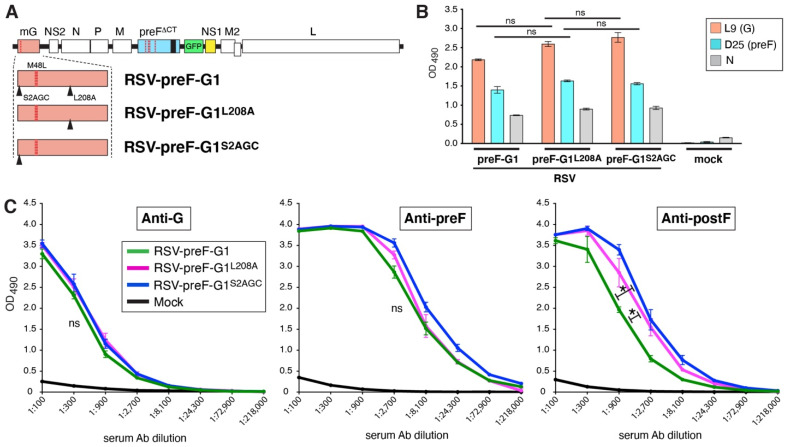
G mutations L208A and S2AGC are not required for induction of high levels of G Abs. (**A**) Genome composition of RSV-preF-G1^L208A^, in which Serine 2 was restored to TCC, and RSV-preF-G1^S2AGC^, in which G residue 208 was restored to leucine. The dotted red lines indicate mutation L208A. Arrowheads indicate the locations of S2AGC and L208A. (**B**) Expression of preF and G proteins on the surface of virus infected cells. Cells were infected with RSV-preF-G1, RSV-preF-G1^L208A^, RSV-preF-G1^S2AGC^ or mock-infected, and G and preF surface levels at 26 hpi were determined using cell ELISA, as described above. (**C**) Serum levels of G-, preF-, and postF-specific IgG Abs induced in vaccinated mice. Three-fold dilutions of pooled boost sera from mice (*n* = 5/group) were examined by ELISA, as described in Figure 2. Error bars represent the standard deviation of the mean of triplicate samples. In (**C**), only statistical differences between RSV-preF-G1^L208A^ and RSV-preF-G1, and between RSV-preF-G1^S2AGC^ and RSV-preF-G1, were determined (* *p* < 0.05; ns, non-significant).

In Figure 6, we found that RSV-preF-G1 did not express higher steady-state levels of G protein in vitro than RSV-preF^∆CT^. RSV-preF-G1 did, however, have higher G levels in the virion, which may have contributed to the improved mouse serum anti-G Ab levels. Other potentially contributing parameters were mutations in G (L208A and the change of ser2 to AGC) and the altered position of NS1. The latter we cannot directly address, because a control virus would have to have both NS1 and G at first position. We did, however, examine the impact of the G mutations. To do so, RSV-preF-G1 was modified to revert the codon for ser2 to the original TCC or to revert residue 208 to leucine (Figure 7A). The resulting viruses, RSV-preF-G1^L208A^ and RSV-preF-G1^S2AGC^, respectively, were then subjected to the same cell ELISA and in vivo Ab analyses (Figure 7B,C). In short, viruses RSV-preF-G1^S2TCC^ and RSV-preF-G1^L208^ expressed very similar levels of G and preF protein in vitro (Figure 7B), and did not change the level of anti-G Ab induction in mice, relative to RSV-preF-G1 (Figure 7C). RSV-preF-G1^S2TCC^ and RSV-preF-G1^L208^ did each induce a minor but significant increase in anti-postF Abs (*p* < 0.05) for reasons not understood.

### 3.8. In Vitro Neutralization

**Figure 8:**
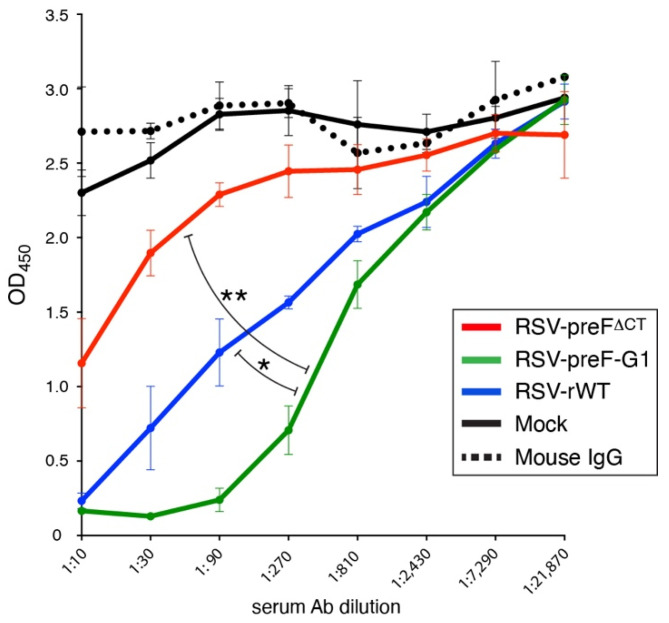
Neutralization of wt RSV in vitro. Neutralization capacity of heat-inactivated mouse sera was determined using wt RSV engineered to express horse-radish peroxidase (RSV-HRP), as described previously [18]. Random mouse IgG and sera from mock-vaccinated mice were included as negative controls. The data points represent the mean of triplicate samples of pooled sera from five mice/group. Error bars represent the standard deviation of the mean. The experiment was performed twice from two independent mouse studies with *n* = 5 each, with similar results. Statistical, differences were determined between RSV-preF-G1 and RSV-rWT, and between RSV-preF-G1 and RSV-preF^ΔCT^ (* *p* < 0.05; ** *p* < 0.01). Curve-fit analyses were performed using Prism9, and reciprocal titers that achieved 50% neutralization of RSV-HRP were calculated (see text). Note that a curve fit for RSV-preF^∆CT^ could not be performed due to incomplete neutralization.

In previous work [18], we found that IN immunization with virus RSV-preF^∆CT^ only partially neutralized a wt virus in vitro. In showing significantly higher anti-G Ab levels than RSV-preF^∆CT^, RSV-preF-G1 displayed potential for improved efficacy. We therefore compared these two viruses to assess the neutralization potential of serum Abs induced by RSV-preF-G1(Figure 8). As a read-out, we used a virus expressing HRP (RSV-HRP), as described previously [18). The pooled sera from the vaccinated mice were heat-inactivated, incubated with RSV-HRP (without added complement), and then used to infect HEp-2 cells. At 48 hpi, the medium was replaced with ELISA substrate and the OD_450_ was determined. The results confirmed the previous finding of partial neutralization of a wt virus by sera from RSV-preF^∆CT^ vaccinated mice. Due to the partial neutralization, a reliable titer for 50% neutralization could not be determined for RSV-preF^∆CT^. In contrast, sera from RSV-preF-G1 vaccinated mice neutralized significantly better, and outperformed sera from RSV-rWT vaccinated mice (reciprocal dilutions for 50% neutralization of 745 and 180, respectively).

### 3.9. Protection from Lung Pathology after Challenge

**Figure 9:**
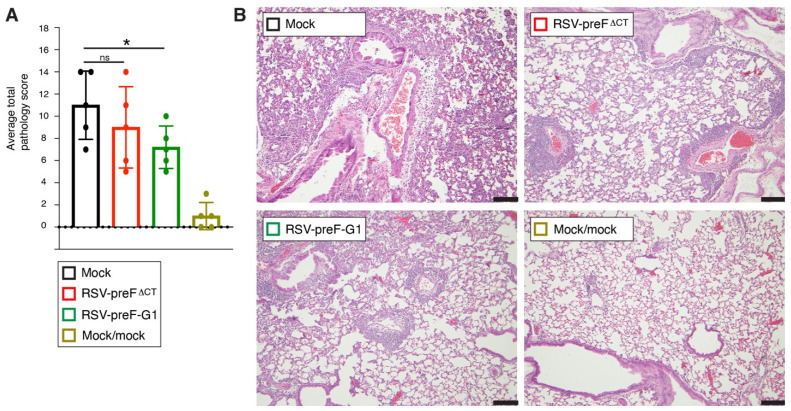
Protection from lung pathology after challenge with wt RSV. Mice were prime-boost vaccinated, as shown in Figure 2B, and challenged at four weeks post-boost with a wt virus containing a line19F mutation (A2/K191R). Five days post-challenge, lungs from challenged mice were collected and processed for histological examination using H&E stain. (**A**) Tissues were examined and scored blindly by an ACVP-certified veterinary pathologist for five parameters commonly assessed for RSV-induced pathology (see text), scoring each parameter from zero (no pathology) to three (high pathology). Mock = mock-vaccinated and challenged. Mock/mock = mock-vaccinated and mock-challenged (mock is supernatant from cells harvested identically to virus stocks). The bars represent the average total pathology score (*n* = 5/group). Error bars represent the standard deviation of the mean (* *p* < 0.05; ns, non-significant). (**B**) Images of H&E stained lung sections, representative of the scores in (**A**); Magnification 100×. Black sizebar: 100 µm.

We previously reported that vaccination with RSV-preF^∆CT^ and surrogate wt virus RSV-rWT substantially and equally reduced lung pathology after challenge with our laboratory wt RSV A2 strain [18]. Because protection by RSV-preF^∆CT^ was already near-complete, and we aimed to determine whether RSV-preF-G1 demonstrated improved efficacy compared to RSV-preF^∆CT^, we used a high dose of a relatively high-pathogenic RSV termed A2/K191R. A2/K191R was generated by incorporating into our lab A2 strain an F mutation (K191R) from line19F RSV, an isolate previously shown to be more pathogenic [39]. Comparing A2/K191R to our regular wt A2 strain, these viruses were used to infect mice, and their weight loss was monitored as an indicator of disease severity (Figure S2). A2/K191R induced significantly higher weight loss than A2, indicating that it was indeed more pathogenic. Next, the mice were vaccinated with RSV-preF^∆CT^, RSV-preF-G1, or mock-vaccinated (mock = uninfected Vbac cells processed identically to virus stocks). Following a prime-boost, as shown in Figure 2B, animals were challenged at four weeks post-boost with 2 million PFU of A2/K191R (Figure 9). An additional group was mock-vaccinated and mock-challenged (Mock/mock). Five days post-challenge, pathology in the lungs was scored blindly by an ACVP-certified pathologist using six parameters commonly assessed: perivascular cuffing (leukocytes surrounding blood vessels), peri-bronchiolar cuffing (space and fluid surrounding bronchioles), interstitial pneumonia (thickness of alveolar septa; leucocytes in the alveolar space), eosinophil influx, mucus, and edema, scoring each parameter from zero (no pathology) to three (high pathology). Mock-vaccinated challenged mice showed an average total pathology score of approximately 11, whereas mock-vaccinated, mock-challenged mice showed an average total pathology score of approximately one. Of the two preF-expressing vaccines, neither was able to fully prevent lung pathology induced by a high dose of A2/K191R. However, RSV-preF-G1 was the only virus that significantly (*p* < 0.05) reduced lung pathology relative to mock-vaccinated challenged mice, thus providing better protection from lung pathology than its parent vaccine RSV-preF^∆CT^. Previous work shows that RSV-preF^∆CT^ and the RSV-rWT vaccination provided equal protection against lung pathology, with a regular A2 strain [18]; this fact suggests that RSV-preF-G1 is more effective than a wt RSV in inducing a protective response.

## 4. Discussion

### 4.1. Study Objective

The objective of this study was to increase the anti-G Ab levels induced by a live single-cycle preF-expressing vaccine, RSV-preF^ΔCT^, in order to increase efficacy and strengthen/broaden the humoral response. While the RSV-preF^ΔCT^ vaccination protected mice from challenge [18], and offers unique features to improve safety and efficacy of a live human RSV vaccine, the vaccine did not induce Abs that neutralized RSV in vitro as well as Abs induced by a wt virus, and it failed to yield significant levels of anti-G Abs. This was in spite of expressing wt levels of G protein at the surface of infected cells in vitro. Serum anti-G Abs may contribute greatly to efficacy and protection against RSV pathology (for review see [23]) and, when co-present with anti-F Abs, broaden the humoral response and may reduce the emergence of viral escape variants. Thus, an examination regarding whether we could enhance the anti-G Ab response was the most important first step, to improve the potential of our single-cycle vaccine for future human application.

### 4.2. Several Modifications to RSV-preF^ΔCT^ Failed to Enhance the Induction of Serum Anti-G Abs in Mice

Mutating residue L208 to alanine, which was reported to prevent G cleavage in Vero cells [11] did not result in a significant change in RSV-preF^ΔCT^-induced anti-G Ab levels (Figure 2). Although the data clearly show that premature CatL cleavage was not a cause of low anti-G Ab levels after vaccination with RSV-preF^∆CT^, G input levels of this vaccine are low and we cannot exclude a more substantial impact of L208A if the input G protein levels were higher. In addition, G is probably not cleaved by CatL in mice, further reducing the likelyhood of observing differences between RSV-preF^∆CT^ and RSV-preF-G^L208A^. Thus, any impact of L208A may be more substantial for inactivated RSV vaccines produced in Vero cells or other cells with high CatL activity. Restoring sG expression in RSV-preF^∆CT^ did not increase anti-G Ab induction either (Figure 3). These data do not exclude the possibility that the inclusion of sG provides some benefit in live vaccines. For example, hamsters were protected better against viral load in the nasal turbinates after vaccination with a parainfluenza virus expressing wt G versus mG alone [17]. However, there is also potential for detrimental host immune impact by sG [13,14,15], and the balance of detrimental versus protective impacts remains unclear. A third approach, presenting preF and G on separate particles in a combination vaccine, also failed to meet our objective (Figure 4). The rationale behind this approach was to question whether we could focus the response on G by removing F from the particle, although some believe there is potential for the opposite, i.e., the shielding of F by the highly glycosylated G protein. In our case, the G Ab levels were not increased by separately presenting the G and preF antigens and the level of anti-preF Abs was moderately reduced, suggesting this strategy is not beneficial.

### 4.3. Addition of Exogenous (Input) G and Moving G to the First Genome Position of RSV-preF^∆CT^ Each Substantially Improve Serum Anti-G Ab Levels in Mice

When we increased the amount of input G by providing exogenous G via a VLP, we observed, for the first time, a significant increase in anti-G Abs (Figure 5). This suggests that the previously observed low level of G in RSV-preF^∆CT^ virions may be a cause of the failure to induce anti-G Abs, and that adding bulk input G protein may rescue this deficit. However, a combined, live-attenuated/VLP vaccine is not likely to be a cost-effective approach from a manufacturing standpoint. In a final approach therefore, we made an effort to raise the G expression levels without exogenous G, by swapping the G and NS1 genes to place G at the first position (Figure 6). Unexpectedly, cell ELISA showed that the level of G at the surface of infected cells was not increased. Nevertheless, vaccination with RSV-preF-G1 yielded a strong increase in anti-G Ab levels, showing that we may also induce both anti-preF and anti-G Abs without the addition of exogenous G protein.

The impact of exogenous G may or may not be a result specific to our single-cycle vaccine. If so, this could potentially be explained by a low rate of RSV-preF^∆CT^ infection or replication in vivo. In this way, in the context of low level replication, input G might have an exaggerated contribution to anti-G Ab induction. However, this explanation would not be consistent with the observed high levels of G and F -specific T cells induced by RSV-preF^∆CT^ [18]. In previous work with another single-cycle virus in which the M gene is absent (RSV-Mnull), M was the only protein not de novo synthesized (due to its gene deletion), and also the only viral protein that lacked a substantial T cell response [36]. This agrees with the dogma that replication and de novo protein synthesis are required for strong T cell development, and suggests that RSV-preF^∆CT^ replicates to sufficiently high levels and that, therefore, low genomic replication is not a main cause of poor anti-G Ab induction. Further modifications to RSV-preF-G1 (Figure 7) confirmed the findings of Figure 2 in that the status of L208 does not impact the anti-G Ab levels either. One variable that we did not examine for impact is the placement of NS1 in eighth position. In the absence of an anti-NS1 Ab, and because this was not the focus of the present study, we did not measure NS1 levels in infected cells. This level is, however, expected to be lower than when expressed from the first genome position, which may have downstream consequences, since NS1 is known to counter the interferon response (for review see [20]). However, in our lab, wt and surrogate wt RSV induce strong anti-G Ab levels with NS1 in the native position [18]). Therefore, the placement of NS1 in its native position is not likely to be responsible for the inability of RSV-preF^∆CT^ to induce high levels of anti-G Ab induction in mice.

The results obtained with virus RSV-preF-G1 provide perhaps the most compelling potential cause for poor anti-G Ab induction by RSV-preF^∆CT^. We determined that RSV-preF-G1 virions contained higher levels of G than did RSV-preF^∆CT^ virions (Figure 6C). Since RSV-preF^∆CT^ and RSV-preF-G1 showed equal G steady-state levels at the infected cell surface in vitro, it is possible that the expression of G from the first position leads to more virion incorporation through a temporal or other unknown effect on G expression or processing. In any case, containing additional G in the virion constitutes a higher level of input G protein, which may have contributed to higher anti-G Ab levels independent of viral replication. Alternatively, increased G virion levels may have altered the rate of infection or uptake by immune cells in vivo, and may have led to an increased level of (replication-dependent) de novo G synthesis or presentation.

### 4.4. In Summary

In this project, we aimed to improve the efficacy of a single-cycle preF-expressing virus, which was protective in mice but failed to induce sufficient levels of anti-G Abs in vivo or to fully neutralize wt RSV in vitro. Only one of four genetic approaches succeeded in enhancing serum anti-G Ab levels. The most successful vaccine (RSV-preF-G1) induced Abs that outperformed a wt RSV in an in vitro neutralization assay and significantly reduced lung pathology in mice challenged with a high dose of pathogenic wt RSV, whereas its parent vaccine (RSV-preF^∆CT^) did not. In an effort to explain the mechanism underlying the efficacy improvement, we excluded a number of potential causes. Currently, we suspect that an increase in virion-associated G protein may have been responsible for enhanced efficacy and the anti-G Ab levels induced by RSV-preF-G1, either by increasing bulk input G, or indirectly, by causing an increase in de novo G synthesis in vivo. In the near future, we plan to further pinpoint the responsible parameters, so that this could potentially be applied universally to live RSV vaccines. Additionally, we hope to study the impact of NS1 re-arrangement in the context of a live single-cycle RSV vaccine, to determine whether this may provide additional vaccine benefits to the single-cycle vaccine approach.

## Data Availability

Not applicable.

## Supplementary Materials

The following supporting information may be downloaded at: https://www.mdpi.com/article/10.3390/v14112474/s1, Figure S1: Validation of Ab ELISA assay; Figure S2: Weight loss induced by challenge virus A2/K191R.
