# Supplementary material for: Enhancing Anti-G Antibody Induction by a Live Single-Cycle Prefusion F—Expressing RSV Vaccine Improves In Vitro and In Vivo Efficacy
Source: Viruses. 2022 Nov 9;14(11):2474. doi: 10.3390/v14112474 (PMC9697216; doi:10.3390/v14112474)
Supplement: Supplementary file 1 [file viruses-14-02474-s001.zip › viruses-1918138-supplementary.pdf]

Supplementary Materials

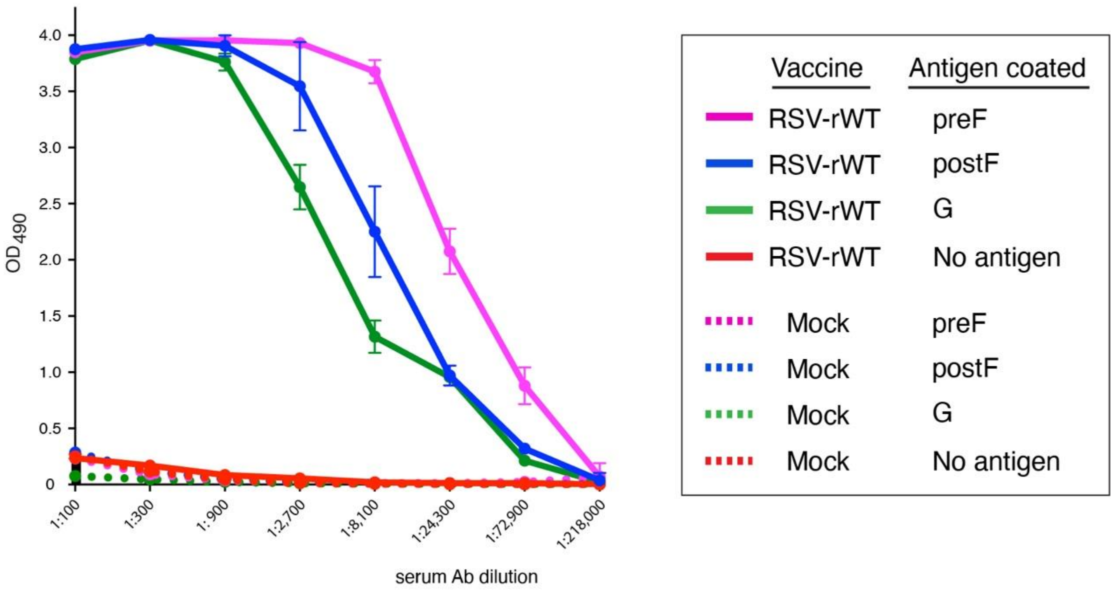

Figure S1. Validation of Ab ELISA assay. Mice were primed and boosted intranasally with 0.5 million PFU of RSV-rWT, a surrogate wildtype virus previously described (18, 35, 36), or mock infected. Pooled sera derived from blood samples taken at 3 weeks post-boost were incubated in a 96 well plate on wells coated overnight with purified preF, postF, or G. The method and the origin of the antigens are described in Materials and Methods. As a negative control, the same sera were incubated on wells that were identically prepared but without antigen. Note that the individual antigens were previously validated (18). Error bars represent standard deviations from the mean of triplicate samples (n=5/group).

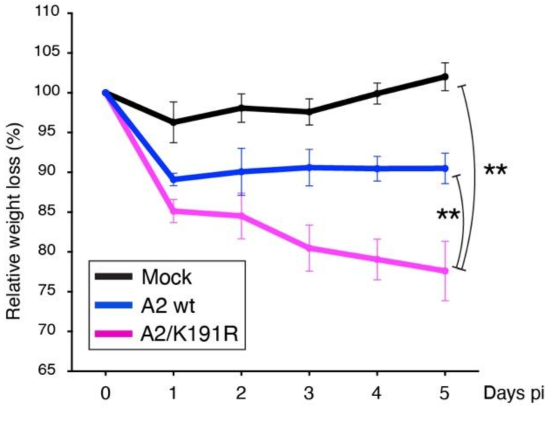

Figure S2. Weight loss induced by challenge virus A2/K191R. Mice were infected intranasally with 2 million PFU of A2/K191R and weight loss was monitored daily. As a comparison, another group of mice was infected with a fully wt A2 RSV (the parent of A2/K191R), or mock-infected as a negative control. Weight loss was expressed as a percentage of the starting weight (set at 100%). Values represent the average of 4 mice/group. Error bars represent the standard deviation of the mean (***p*<0.01).
